# Dual resonance energy transfer in triple-component polymer dots to enhance electrochemiluminescence for highly sensitive bioanalysis†
†Electronic supplementary information (ESI) available: Experimental section, supporting figures and tables. See DOI: 10.1039/c9sc01570a


**DOI:** 10.1039/c9sc01570a

**Published:** 2019-05-30

**Authors:** Ningning Wang, Ziyu Wang, Lizhen Chen, Weiwei Chen, Yiwu Quan, Yixiang Cheng, Huangxian Ju

**Affiliations:** a State Key Laboratory of Analytical Chemistry for Life Science , School of Chemistry and Chemical Engineering , Nanjing University , Nanjing 210023 , China . Email: hxju@nju.edu.cn; b Key Lab of Mesoscopic Chemistry of MOE , Jiangsu Key Laboratory of Advanced Organic Materials , School of Chemistry and Chemical Engineering , Nanjing University , Nanjing 210023 , China . Email: yxcheng@nju.edu.cn

## Abstract

Polymer dots (Pdots) have become a type of attractive illuminant for electrochemiluminescence (ECL). However, the low ECL efficiency severely limits their practicability. Here, we design a dual intramolecular resonance energy transfer (RET) mechanism with newly synthesized triple-component Pdots to achieve great ECL enhancement. This mechanism efficiently shortens the path of energy transmission, thus greatly promoting the ECL amplification by 380 and 31 times compared to systems with no and single RET, and results in a relative ECL efficiency of 23.1% (*vs.* 1 mM Ru(bpy)3^2+^). Using metal–organic frameworks to carry the triple-component Pdots, a highly luminescent probe is proposed. By integrating the probe with target-mediated enzymatic circulation amplification and DNA arrays, a highly sensitive ECL imaging method is designed for simultaneous visual analysis of two kinds of proteins, mucin 1 and human epidermal growth factor receptor 2, on living cells, which exhibited linear ranges of 1 pg mL^–1^ to 5 ng mL^–1^ and 5 pg mL^–1^ to 10 ng mL^–1^ with limits of detection of 1 pg mL^–1^ and 5 pg mL^–1^, respectively. The proposed strategy showed promising application in bioanalysis.

## Abstract 2

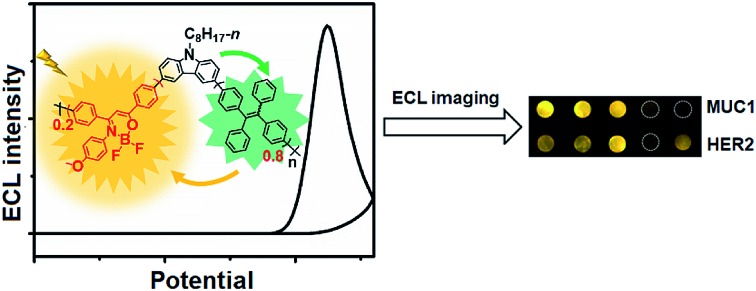
A dual intramolecular electrochemiluminescence resonance energy transfer process is proposed with triple-component Pdots to enhance ECL emission, which greatly improves the ECL efficiency and can be used for sensitive and specific visual quantification of different targets.

## Introduction

Owing to their good biocompatibility, low cellular toxicity, easy functionalization, high photostability, and size/surface-trap controlled luminescence, polymer dots (Pdots) have been widely applied as promising fluorescent bioprobes in single particle tracing, biosensing and cellular labeling.^1^–^6^ The electrochemiluminescence (ECL) of Pdots has also attracted considerable attention due to their tunable optical and electrochemical properties.^7^–^10^ However, the ECL application of Pdots, especially in ECL imaging that requires a higher luminescence intensity, is still a great challenge due to their relatively lower ECL efficiency and/or higher ECL potential than general inorganic emitters.^11^,^12^

To improve the ECL performance of Pdots, some efficient approaches have been developed, such as introducing donor–acceptor (D–A) type^13^,^14^ or aggregation-induced emission (AIE)-active moieties^15^,^16^ into the conjugated polymer backbones. In particular, the AIE-active ECL luminophores have been regarded as one of the most interesting members due to their amplification effect in aggregate states.^17^ Moreover, intramolecular D–A transfer can activate tunable ECL signals, which provides an alternative pathway to improve the ECL response. Inspired by these results, in our previous work we synthesized two kinds of three-component Pdots with the same AIE-active moiety but different D–A structures containing carbazole and fluorene, respectively, and proved the stronger electron-donating ability of the carbazole moiety with a lower ECL onset potential and higher intensity of ECL emission.^18^ To achieve excellent ECL performance, this work further screened optimal types of the acceptor and an appropriate ratio of D/A for the preparation of Pdots, and designed a dual resonance energy transfer (RET) (D1–A1/D2–A2) mechanism to overcome the bottleneck of Pdots in ECL efficiency.

**Scheme 1:**
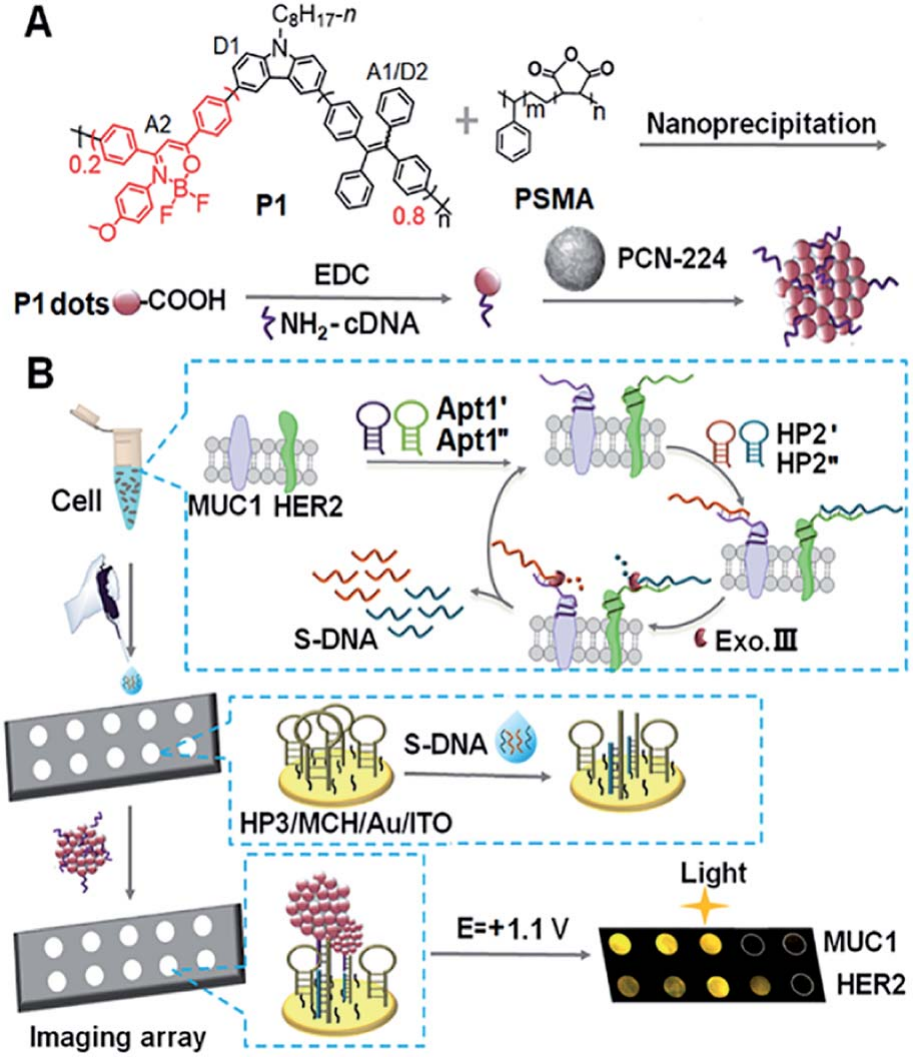
Schematic diagram of (A) preparation of Pdots@PCN-224 as an ECL probe and (B) ECL imaging array for the analysis of two kinds of membrane proteins with target-mediated cyclic amplification.

As well known, ECL–RET from the donor to acceptor, similar to Förster RET (FRET), has demonstrated its ability to extend ECL applications to biosensing^19^,^20^ and monitoring molecular interactions^21^ owing to potential and spatial control.^22^,^23^ By incorporating Ru(bpy)_3_^2+^ into Pdots, the RET from the excited Pdots to encapsulated Ru(bpy)_3_^2+^ can enhance the ECL emission by 15.7 fold.^24^ The intramolecular RET achieved by integrating luminol (donor) and Ru(bpy)_2_(mcpbpy)^2+^ (acceptor) into one molecule has showed enhanced ECL emission owing to the short path of energy transmission and less energy loss.^25^ In this work, triple-component (D1, A1/D2 and A2) Pdots (**P1** dots) are composited with carbazole, tetraphenylethene (TPE), and 2,2-difluoro-3-(4-methoxyphenyl)-4,6-diphenyl-2*H*-1,3l4,2l4-oxazaborinine (DMDO) (Scheme 1A). The carbazole moiety is electroactive and can be oxidized at a relatively low potential to produce the excited state and act as D1. The energy is then transferred to the TPE moiety that acts as both A1 and D2 to transfer the energy to DMDO (A2). The short path of energy transmission greatly promotes ECL amplification for the preparation of the sensitive ECL probe. By integrating the probe with target-mediated enzymatic circulation amplification and DNA arrays (Scheme 1B), the **P1** dots have successfully been used for highly sensitive ECL imaging. Therefore, a simultaneous visual detection method for two kinds of membrane proteins, mucin 1 (MUC1) and human epidermal growth factor receptor 2 (HER2), on living cells was developed with specific recognition and DNA coding technology. The excellent performance of the proposed method indicates the promising application of the dual RET mechanism along with the triple-component Pdots in bioanalysis.

## Results and discussion

### Design and characterization of **P1–P4** and Pdots

**Fig. 1:**
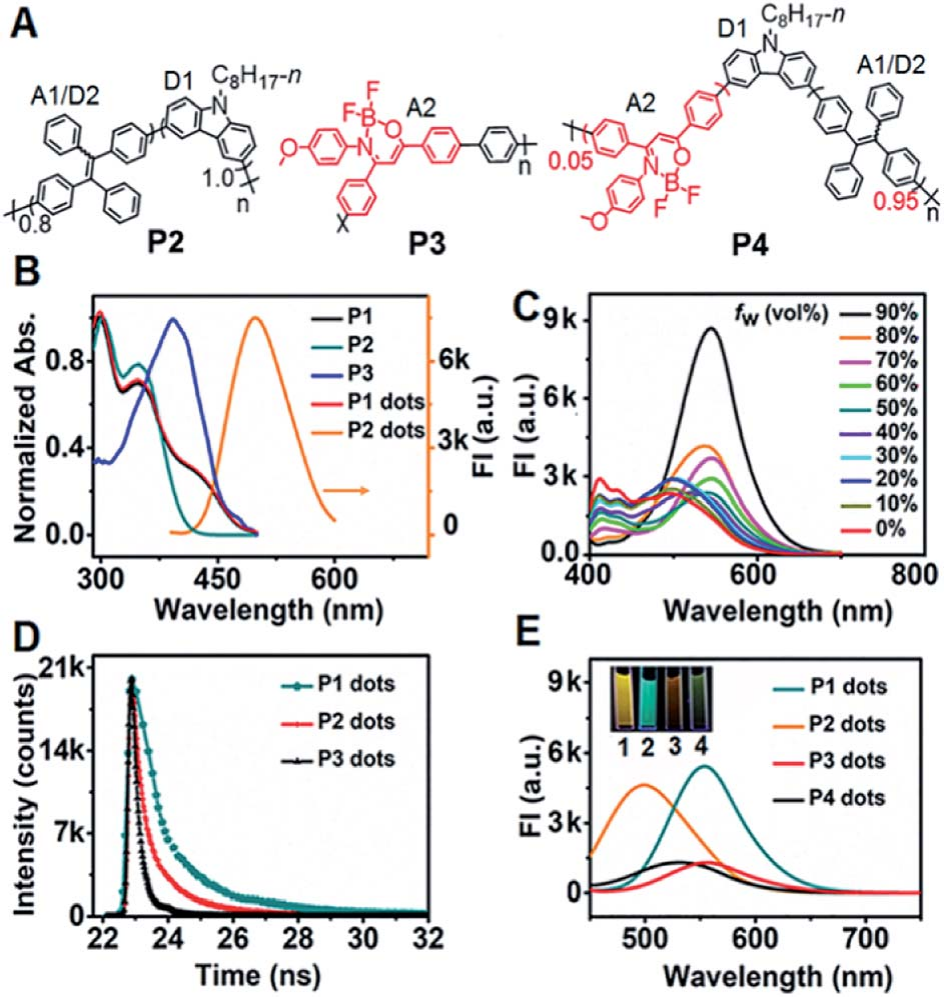
(A) Structural formulae of **P2–P4**. (B) UV-vis absorbance of **P1–P3** in THF and **P1** dots in H_2_O, and the FL spectrum of **P2** dots in H_2_O at a *λ*_ex_ of 370 nm. (C) FL spectra of **P1** in THF–H_2_O mixtures with different contents of H_2_O at a *λ*_ex_ of 370 nm. (D) FL lifetimes of **P1–P3** dots in H_2_O at a *λ*_ex_ of 374 nm. (E) FL spectra of 10 μg mL^–1^**P1** dots, 50 μg mL^–1^**P2** dots, 100 μg mL^–1^**P3** dots, and 50 μg mL^–1^**P4** dots at a *λ*_ex_ of 370 nm. Inset: FL photos of **P1–P4** dots.

In the dual intramolecular RET mechanism, AIE-active TPE plays key roles (A1/D2). As the proof-of-concept, three model polymers (**P2–P4**) were designed (Fig. 1A). Specifically, **P2** included the moieties of D1 and A1/D2 with single ECL–RET, while **P3** consisted of A2 without ECL–RET. Besides, **P4** was composed of more A1/D2 and less A2 compared to **P1** and acted as the model of incomplete dual ECL–RET. In **P1**, the optimal ratio of A1/D2 to A2 was 8 : 2 (Fig. S1†). The detailed synthetic routes of **P1–P4** were modified according to our previous reports (Scheme S1†).^26^–^28^ The Pdots could be prepared by a nanoprecipitation method utilizing a polymer as the luminescent precursor and poly(styrene-*co*-maleic anhydride) (PSMA) as a functional reagent.

**P1** exhibited three distinct absorption peaks in THF solution centered at 300, 349 and 429 nm, respectively (Fig. 1B). The peaks at 300 and 349 nm also occurred in the absorption spectrum of **P2**. Compared with **P2** and **P3**, the shoulder absorption peak at 429 nm could be assigned to the DMDO chromophore (A2), indicating the intramolecular charge transfer process from the electron-rich carbazole donor to the electron-deficient DMDO acceptor *via* the TPE bridge linker.^29^ The identifiable overlap of the absorption of **P1** dots with the emission of **P2** dots in H_2_O demonstrated the feasibility of the second FRET process from the TPE moiety (A1/D2) to DMDO (A2) (Fig. 1B).

The fluorescence spectrum of **P1** in THF showed a weak emission situated at 412 nm with a shoulder peak at 433 nm (Fig. 1C), which could be attributed to the carbazole moiety. Upon addition of H_2_O to THF, these peaks gradually decreased and disappeared until the water fraction (*f*_w_) reached 70%. Meanwhile, the peak at 496 nm due to the emission of the TPE moiety gradually increased from *f*_w_ = 0 to 30%, and then disappeared at *f*_w_ = 40%, indicating the first intramolecular FRET process from the carbazole moiety to the TPE moiety.^18^ With increasing *f*_w_, a new emission peak assigned to the DMDO chromophore appeared at 527 nm, which gradually redshifted to 548 nm at a *f*_w_ of 90% with an abrupt increase of the peak intensity at *f*_w_ in the range of 80% to 90%, demonstrating the presence of the second intramolecular FRET process in **P1** dots at high water fractions.

The **P1** dots showed a fluorescence lifetime *τ* of 8.00 ns (CHISQ = 1.03), which was much longer than 1.58 ns of **P2** dots (CHISQ = 1.00) (Fig. 1D), demonstrating the different fluorescent emission moieties and the presence of the second FRET in **P1** dots. The fluorescence lifetime of 2.68 ns (CHISQ = 1.09) for **P3** dots was also slightly longer than that of **P2** dots. Therefore, the fluorescence spectra of **P1** and **P3** dots exhibited different emission peak wavelengths from that of **P2** dots (Fig. 1E). When the fraction of DMDO decreased to 0.05, a weak emission peak appeared at 530 nm, indicating the weakened FRET in **P4** dots due to an incomplete dual RET, which also demonstrated the FRET effect from TPE to DMDO.

### Dual ECL–RET of **P1** dots

**Fig. 2:**
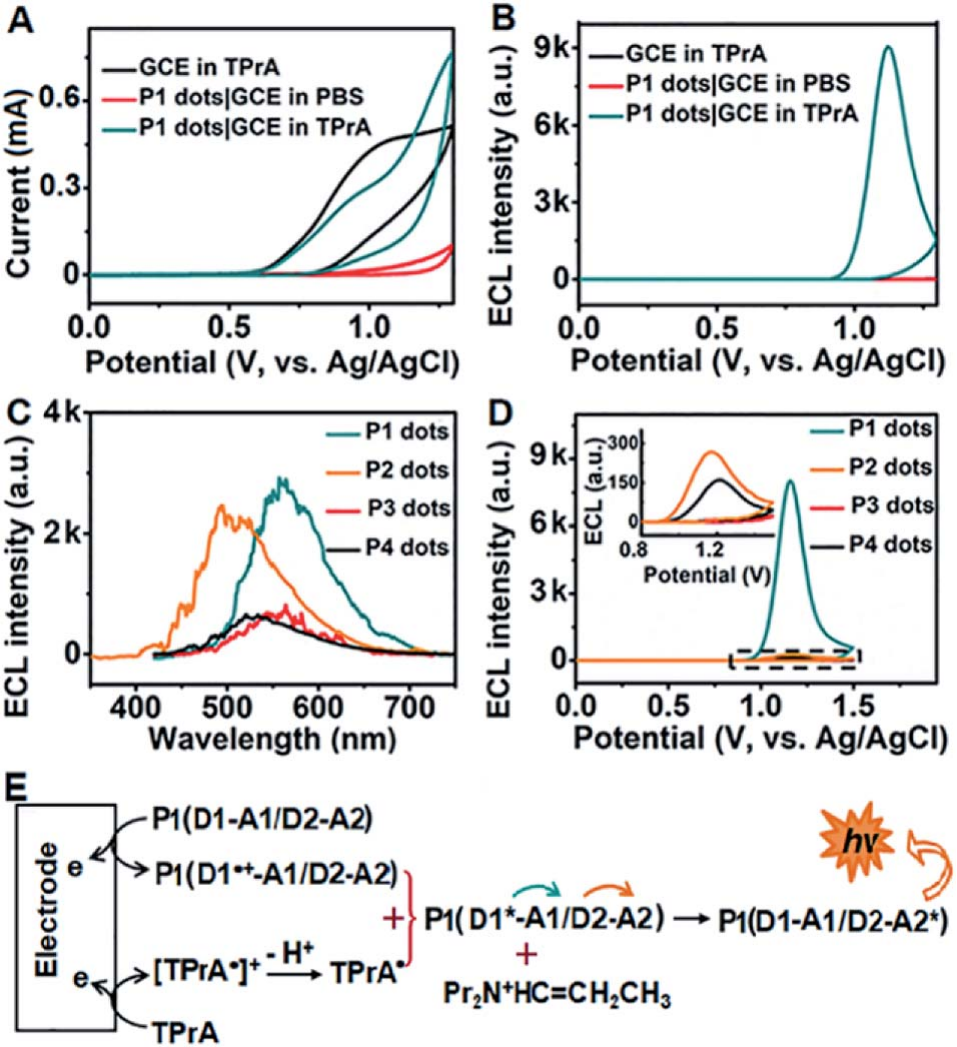
(A) CV and (B) ECL curves of bare and **P1** dot modified GCE in 0.1 M pH 7.4 PBS in the presence and absence of 25 mM TPrA as the co-reactant. (C) ECL spectra of 10 μg mL^–1^**P1** dots, 50 μg mL^–1^**P2** dots, 100 μg mL^–1^**P3** dots, and 50 μg mL^–1^**P4** dots in 100 mM TPrA. (D) ECL curves of 50 μg mL^–1^**P1–P4** dots|GCE in 25 mM TPrA. Inset: enlarged view of the dotted line region. PMT: 300 V. Scan rate: 100 mV s^–1^. (E) ECL mechanism of **P1** dots in TPrA (**P1** represents **P1** dots).

In the presence of tri-*n*-propylamine (TPrA) as a co-reactant, a **P1** dot modified glassy carbon electrode (GCE) showed the maximum ECL intensity, compared with the responses in the presence of other coreactants such as K_2_C_2_O_4_, Na_2_SO_3_ and TEA (Fig. S2†). The ECL intensity increased with increasing concentration of TPrA and then decreased to a steady value at 25 mM (Fig. S3†). Thus 25 mM TPrA was chosen as the optimal amount of the anodic co-reactant. TPrA showed a broad irreversible oxidation peak at +1.02 V with an onset potential of +0.57 V at the GCE (Fig. 2A).^30^ Meanwhile, **P1** dots|GCE in PBS showed an oxidation current, indicating that **P1** dots were electrochemically oxidized to form cationic radicals by injecting holes into the highest occupied molecular orbital.^31^ As a result, the ECL emission of **P1** dots|GCE occurred at +1.12 V with the onset potential of +0.89 V in the presence of TPrA (Fig. 2B). The ECL spectra of **P1–P4** dots coincided well with their fluorescence spectra (Fig. 2C), indicating the presence of the same excited species in ECL emission as those in fluorescence emission.^32^ Similar to the FRET enhancement in fluorescence emission, the enhanced ECL emission of **P1** dots|GCE compared with **P2–P4** dots|GCE (Fig. 2D) was caused by the RET process. Moreover, the onset potential of +0.89 V for **P1** dots|GCE was similar to that of **P2** dots (+0.87 V) and **P4** dots (+0.93 V) but much lower than that of **P3** dots (+1.13 V) (Fig. 2D). These phenomena were further evidence of the detailed mechanism of dual RET in **P1** dots (Fig. 2E): TPrA was first oxidized and deprotonated to produce a reducing agent (TPrA˙) at potentials more positive than +0.57 V. Afterward, the electrons were injected into oxidized Pdots˙^+^ (D1˙^+^–A1/D2–A2), which produced the excited Pdots* (D1*–A1/D2–A2), and the energy was transferred to TPE and then to DMDO to generate excited D1–A1/D2–A2* for ECL emission. As a result, the ECL intensity for **P1** was about 31- and 380-fold higher than those of single (**P2**) and no RET (**P3**) systems, respectively (Fig. 2D).

The relative ECL efficiency of 50 μg mL^–1^**P1** dots *vs.* 1 mM Ru(bpy)_3_^2+^ was calculated to be 23.1% (Fig. S4†), which was obviously higher than those of commercial poly[2-methoxy-5-(2-ethyl hexyloxy)-1,4-(1-cyanovinylene-1,4-phenylene)] Pdots (11.22%)^12^ and previously reported triple-component Pdots (Table S1†). The ECL emission of **P1** dots also showed the lowest onset and peak potentials, compared with several commercial Pdots (Fig. S5 and Table S2†), demonstrating the distinguished performance of **P1** dots in ECL emission.

To assess the intrinsic energy parameters and infer the relative stability of electron/hole injected **P1** dots, annihilation ECL was performed in the absence of TprA. As expected, **P1** dots could be electrochemically oxidized to form cation radicals or reduced to anion radicals in PBS by injecting holes or electrons into **P1** dots (Fig. S6A†),^33^ which led to a strong annihilation ECL emission with a peak at +1.07 V during anodic scanning, and a weak ECL signal in the cathodic region with an onset potential of –1.30 V, respectively (Fig. S6B†). No matter in the oxidative or reductive process-initiated ECL transients, distinguished ECL only appeared at +1.10 V (Fig. S6C and D†), demonstrating that the radical for the anodic ECL emission was more stable.^34^ In addition, considering that the same excited species are present in both annihilation and co-reactant ECL emission, the ECL band gap of **P1** dots could be estimated from the onset potentials of anodic and cathodic annihilation ECL emission at +0.84 and –1.30 V to be 2.14 eV (Fig. S6B†). This was consistent with the energy level (2.16 eV) from CV of **P1** that gave quasi-reversible oxidation peaks with an onset potential of +0.78 V resulting from the oxidation of the TPE moiety^35^ and an onset reduction potential of –1.38 V (Fig. S7†).

### Target-mediated enzymatic circulation amplification

**Fig. 3:**
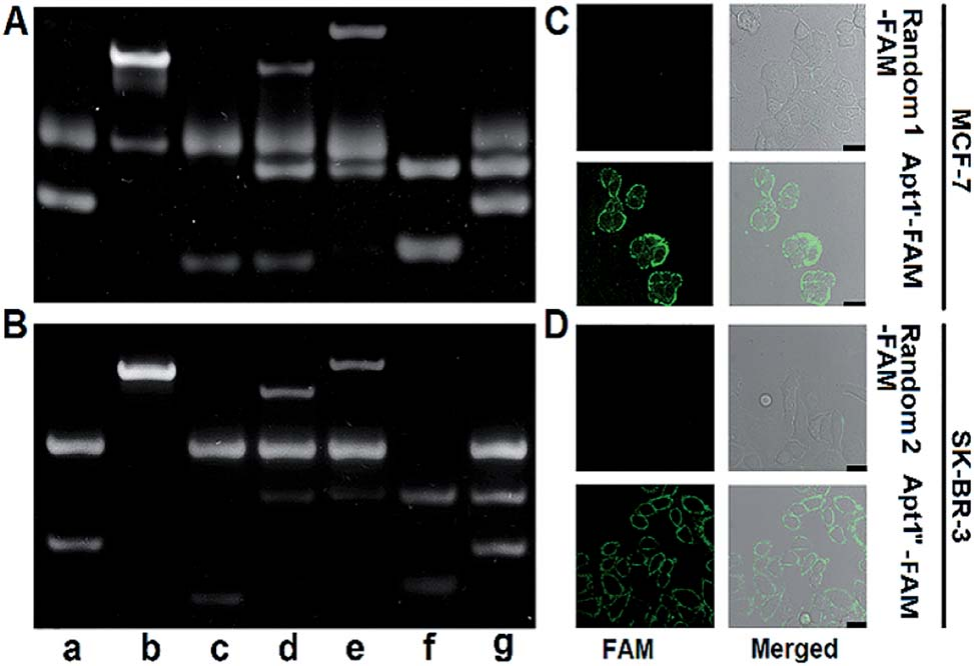
PAGE images for verifying the feasibility of (A) MUC1 and (B) HER2-mediated enzymatic circulation amplification. (a) Apt1 + HP2, (b) (a) + MUC1 or HER2, (c) (b) + Exo III, (d) (c) deactivated at 85 °C for 10 min + HP3, (e) (d) + cDNA, (f) HP3 + cDNA, and (g) Apt1 + HP2 + HP3. CLSM images of (C) MCF-7 cells after incubation with 1 μM random 1-FAM and Apt1′-FAM, and (D) SK-BR-3 cells after incubation with 1 μM random 2-FAM and Apt1′′-FAM. Scale bars: 25 μm.

The designed **P1** dots with a high ECL efficiency showed very low cytotoxicity (Fig. S8†), providing promising application in bioanalysis. Using two kinds of tumor-associated membrane proteins, MUC1 and HER2, as model targets, Apt1 for coding the targets by the specific recognition of aptamers to proteins, HP2 for performing target-mediated enzymatic circulation amplification, HP3 for capturing the released codes (S-DNA) on a DNA array, and capture DNA (cDNA) for labelling the ECL probe (**P1** dots@PCN-224), a sensitive ECL imaging platform was developed. The detailed DNA sequences are listed in Table S3.† As shown in Fig. 3A and B, the recognition of aptamers to proteins induced the hybridization of Apt1 with HP2 (lines a and b), which produced a band at low migration, and exonuclease III (Exo III) could digest the Apt1/HP2 duplex to produce the S-DNA with a band at high migration (line c), which hybridized with HP3 to form a new band (line d). The hybridization product of S-DNA and HP3 could be recognized by cDNA to form another band at relatively low migration (line e). These phenomena verified the feasibility of the designed imaging strategy, including coding, amplification, and capture processes. Moreover, the hybridization between HP3 and cDNA, as well as among Apt1, HP2 and HP3, in the absence of the target could be excluded (lines f and g).

The specific recognition of Apt1 toward targets was further demonstrated with confocal laser scanning microscopy (CLSM) imaging on the living cell surface (Fig. 3C and D). Compared to the treatment with random 1-FAM and random 2-FAM, bright fluorescence was observed on both MUC1-over-expressed MCF-7 cells^36^ upon incubation with Apt1′-FAM and HER2-over-expressed SK-BR-3 cells^37^ upon incubation with Apt1′′-FAM, respectively.

### Characterization of the **P1** dots@PCN-224 probe

**Fig. 4:**
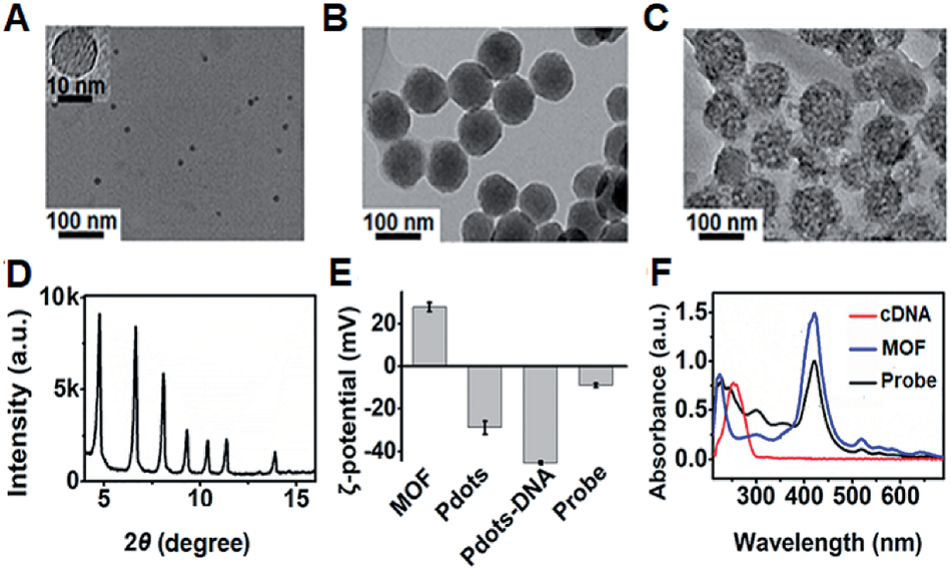
TEM images of (A) **P1** dots, (B) PCN-224 NPs, and (C) probe. (D) PXRD pattern of MOF nanoparticles. (E) *ζ*-Potential characterization. (F) UV-vis absorbance of MOF NPs, cDNA, and the probe.

The transmission electron microscopy (TEM) images of **P1** dots and metal–organic framework (MOF) nanoparticles (PCN-224) showed the spherical and monodisperse feature with an average diameter of around 11 nm (Fig. 4A) and 83 nm (Fig. 4B), respectively. The ECL probe was prepared with PCN-224 as the carrier to assemble the cDNA functionalized **P1** dots through electrostatic adsorption (Scheme 1A), which could further enhance the ECL emission. Morphologic characteristics also demonstrated the successful assembly of the probe, which showed good distribution and dense loading of **P1** dots on PCN-224 (Fig. 4C). Moreover, the **P1** dots@PCN-224 probe could maintain a stable hydrodynamic diameter (Fig. S9†), which proved the good stability of the probe. In addition, powder X-ray diffraction (PXRD) data demonstrated the pure phase of PCN-224 (Fig. 4D), and positively charged PCN-224 (ref. 38) could easily adsorb negatively charged **P1** dots–DNA (Fig. 4E). After loading **P1** dots–cDNA, the MOFs exhibited two new peaks at 247 and 358 nm (Fig. 4F), attributed to the merging of MOF absorption around 224 nm with cDNA absorption at 255 nm, and the absorption of **P1** dots at 348 nm (Fig. 1B), respectively.

### Protein profiling and cell identification

The assembly process of DNA sequences on Au/ITO for ECL imaging was examined with electrochemical impedance spectroscopy (Fig. S10†). Upon the self-assembly of HP3 and then blocking with 6-mercapto-1-hexanol (MCH) on Au/indium tin oxide (ITO), the electron-transfer resistance (*R*_ct_) successively increased. After incubating with S-DNA, the *R*_ct_ further increased, indicating the successful capture of the released S-DNA in the target recycling mixture.

**Fig. 5:**
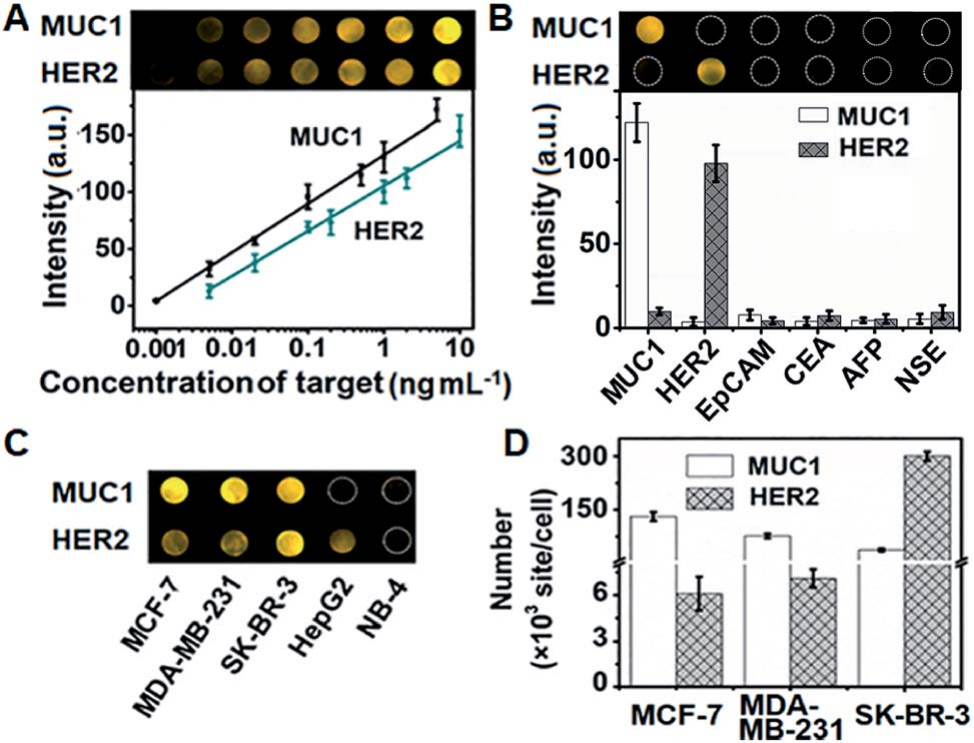
(A) ECL image and calibration curves for the detection of MUC1 and HER2. (B) Specificity of the proposed strategy for imaging detection of MUC1, HER2, EpCAM, CEA, AFP and NSE at 1 ng mL^–1^. (C) ECL image and (D) detection results of MUC1 and HER2 on different cells (*n* = 3).

In order to achieve highly sensitive imaging, the Apt1 concentration, digestion time, amount of Exo III, HP3 concentration and incubation time of the probe were optimized, sequentially (Fig. S11†). Under the optimized detection conditions, the probe was introduced into the DNA array for ECL imaging measurements through the hybridization of cDNA with open HP3. With increasing concentration of MUC1 and HER2, the ECL brightness increased correspondingly and depended linearly on the logarithm of the protein concentration in the range of 1 pg mL^–1^ to 5 ng mL^–1^ for MUC1 and 5 pg mL^–1^ to 10 ng mL^–1^ for HER2 with limits of detection (LODs) of 1 pg mL^–1^ and 5 pg mL^–1^ at a signal-to-noise ratio of 3, respectively (Fig. 5A). The sensitive strategy based on triple amplification processes showed more excellent performance compared with several previous reports (Table S4†). The ECL responses to different proteins such as the epithelial cell adhesion molecule (EpCAM), carcinoembryonic antigen (CEA), alpha fetoprotein (AFP), neuron-specific enolase (NSE), MUC1 and HER2 demonstrated that the proposed imaging method exhibited high specificity to the target proteins (Fig. 5B).

The proposed strategy could be effectively extended to the quantification of membrane proteins on various types of cells. It is well known that HepG2 cells do not express MUC1, and NB-4 cells do not also overexpress MUC1 and HER2. Thus the ECL image showed obviously different responses to three kinds of breast cancer cells (MCF-7, MDA-MB-231 and SK-BR-3) (Fig. 5C). The average amounts of MUC1 on each MCF-7, MDA-MB-231 and SK-BR-3 cell were determined to be 13.1, 7.64 and 3.71 × 10^6^ sites, while the average amounts of HER2 on these cells were 6.1, 7.1 and 301 × 10^5^, respectively (Fig. 5D). These results were close to the data reported previously.^39^,^40^ The relative standard deviation for three parallel measurements was less than 12.2% (Fig. S12†), indicating the high specificity and good accuracy of the imaging strategy.

## Conclusions

A dual intramolecular RET mechanism is proposed with newly designed triple-component **P1** dots to greatly enhance the ECL emission of Pdots. The **P1** dots contain two pairs of energy donors and energy acceptors, which reduce the path of energy transmission and energy loss, and thus show a high ECL efficiency at a relatively low ECL potential. Through loading DNA functionalized **P1** dots on MOFs as a carrier, a sensitive ECL probe has been structured for highly sensitive bioanalysis by the introduction of target-mediated enzymatic circulation amplification as the third amplification process. The proposed ECL imaging platform shows excellent performance for sensitive and specific quantification of cell surface MUC1 and HER2 with an LOD in the pg mL^–1^ level, and can be used for screening of breast cancer cells. By using different aptamers to specifically code the targets, the designed method can be conveniently extended for simultaneous visual analysis of different targets. This dual intramolecular RET strategy provides a new concept for ECL enhancement, and broadens the application of Pdots in ECL imaging and bioanalysis.

## Conflicts of interest

The authors declare no competing financial interests.

## Supplementary Material

Supplementary information
