# Supplementary material for: Dual resonance energy transfer in triple-component polymer dots to enhance electrochemiluminescence for highly sensitive bioanalysis
Source: Chem Sci. 2019 May 30;10(28):6815–20. doi: 10.1039/c9sc01570a (PMC6657406; doi:10.1039/c9sc01570a)
Supplement: Supplementary file 1 [file SC-010-C9SC01570A-s001.pdf]

Electronic Supplementary Material (ESI) for Chemical Science.
This journal is © The Royal Society of Chemistry 2019

Supporting Information

Dual resonance energy transfer in triple-component polymer dots to enhance electrochemiluminescence for highly sensitive bioanalysis

Ningning Wang,[a] Ziyu Wang,[b] Lizhen Chen,[a] Weiwei Chen,[a] Yiwu Quan,[b] Yixiang Cheng*[b] and Huangxian Ju*[a]

[a]State Key Laboratory of Analytical Chemistry for Life Science, School of Chemistry and Chemical Engineering, Nanjing University, Nanjing 210093, P.R. China.

[b]Key Lab of Mesoscopic Chemistry of MOE and Jiangsu Key Laboratory of Advanced Organic Materials, School of Chemistry and Chemical Engineering, Nanjing University, Nanjing 210023, P.R. China.

E-mail: hxju@nju.edu.cn; yxcheng@nju.edu.cn.

Contents

Experimental Section S3

Materials and Reagents S3

Apparatus S3

Synthesis of P1-P4 S4

Synthesis of PCN-224 S7

Preparation of Pdots and Pdots-DNA S7

Preparation of Pdots@PCN-224 Probe S7

Polyacrylamide Gel Electrophoresis (PAGE) Analysis S7

Cell Culturing S7

CLSM Imaging of Apt1 Binding to MUC1 and HER2 on Cell Surface S8

Exo III-Assisited Recycling Assay S8

Fabrication of HP3/MCH/Au/ITO Electrode S8

Measurement Procedure S9

ECL Imaging Detection S9

ECL Spectra 9

Supporting Figures S1-S12 S10-S14

Supporting Tables S1-S4 S15-S17

Annex 1H NMR for **P1-P4** S18-S19

Supporting References S20

Experimental Section

Materials and Reagents. Tetrakis(4-carboxyphenyl)porphyrin (H_2TCPP), benzoic acid (BA), zirconyl chloride octahydrate ($ZrOCl_2 \cdot 8H_2O$), tetrahydrofuran (THF), and N,N-dimethylformamide (DMF) were purchased from J&K Chemical Ltd. (Beijing, China). Tris (2-carboxyethyl) phosphine hydrochloride (TCEP), 6-mercapto-1-hexanol (MCH), 1-ethyl-3-(3-dimethylaminopropyl)carbodiimide (EDC), tri-n-propylamine (TPrA), triethylamine (TEA), and poly(styrene-co-maleic anhydride) (PSMA) (average Mn: 1700) were obtained from Sigma-Aldrich Co., Ltd. (Shanghai, China). Poly[(9,9-dioctylfuor-enyl-2,7-diyl)-alt-co-(1,4-benzo-{2,1′,3}-thiadiazole)] (PFBT), poly(9,9-dioctylfluorenyl-2,7-diyl) (PFO), and poly[2-methoxy-5-(2-ethylhexyloxy)-1,4-(1-cyanovinylene-1,4-phenylene)] (CNPPV) were purchased from American Dye Source, Inc. (Quebec, Canada). Indium tin oxide (ITO) glass slides (100 × 100 mm × 1.1 mm) were obtained from Zhuhai Kaivo Optoelectronic Technology Co., Ltd. (Zhuhai, China). Mucin 1 (MUC1) and human epidermal growth factor receptor 2 (HER2) were purchased from Shanghai Anyan Trading Co., Ltd. (Shanghai, China). Exonuclease III (Exo III) was obtained from New England Biolabs Ltd. (Beijing, China). Fetal bovine serum (FBS) and goat serum were purchased from Thermo Fisher Scientific Inc. (USA). MCF-7 cells, SK-BR-3 cells, MDA-MB-231 cells, HepG2 cells, NB-4 cells, trypsin, and cell culture media such as RPMI-1640 and Dulbecco's Modified Eagle's Medium (DMEM) were supplied by KeyGen Biotech Co., Ltd. (Nanjing, China). Oligonucleotides were synthesized and purified by Sangon Bioengineering Co., Ltd. (Shanghai, China).

Ultrapure water obtained from Millipore water purification system (≥18 MΩ cm^{-1}, Milli-Q, Millipore) was used in all assays. Phosphate buffer solution (PBS, 0.1 M, pH 7.4) was prepared by mixing stock solutions of NaH_2PO_4 and Na_2HPO_4. ECL measurements were conducted in 0.1 M pH 7.4 PBS containing 0.1 M KNO_3 as the electrolyte. All other reagents were of analytical grade and from Sinopharm Chemical Reagent Co., Ltd. (Shanghai, China).

Apparatus. Powder X-ray diffraction (PXRD) data was obtained on a Bruker D8-Advance diffractometer with a Cu-sealed tube (λ = 1.54178 Å) at 40 kV and 40 mA with 0.2 s per step. Transmission electron micrographs (TEM) were acquired with JEM-2100 transmission electron microscope (JEOL Ltd., Japan), and ξ-potential analysis was performed on a 90 Plus/BI-MAS equipment (Brookhaven Instruments Co., USA). Fluorescence measurements were performed on

an F-7000 fluorescence spectrophotometer (Hitachi, Japan). UV-vis absorption spectra were obtained on a Nanodrop-2000C UV-vis spectrophotometer (Thermo, USA). Confocal fluorescence micrographs of cells were acquired on a TCS SP5 confocal laser scanning microscope (CLSM) (Leica, Germany). Numbers of cells were calculated using a Countess® II FL Automated Cell Counter (Thermo Fisher Scientific, USA). Native polyacrylamide gel electrophoresis (PAGE) was performed on an Electrophoresis Analyzer (Bio-Rad, USA) and imaged on a Bio-Rad ChemDoc XRS facility (Bio-Rad, USA). Electrochemical experiments were performed on a CHI 660D electrochemical workstation (CHI instruments Inc., China), while ECL experiments were carried out in a self-made cell on MPI-A multifunctional electrochemical and chemiluminescent analytical system (Xi'an Remex Analytical Instrument Co., Ltd. China). Electrochemical impedance spectroscopic (EIS) measurements were performed on a PGSTAT30/FRA2 system (Autolab, the Netherlands) in 0.1 M KCl aqueous solution containing 5 mM $K_3Fe(CN)_6/K_4Fe(CN)_6$ (1:1). ECL images were captured by a self-made multicolor ECL imaging system equipped with focus lens (EF 50mm f/1.2L USM, Canon) and Retiga R6 color scientific CCD camera (QImaging, Canada) in a dark box.

Synthesis of P1-P4. Three monomers 9-octyl-3,6-bis(4,4,5,5-tetramethyl-1,3,2-dioxabo-rolan-2-yl)-9H-carbazole (**M-1**), 4,6-bis(4-bromophenyl)-2,2-difluoro-3-(4-methoxyphenyl)-2H-1,3l4,2l4-oxazaborinine monomer (**M-2**) and 1,2-bis(4-bromophenyl)-1,2-diphenylethene (**M-3**) were synthesized *via* the method described previously.[S1-S3] The synthesis routs of **M-1**, **M-2**, and **P1-P4** were outlined in Scheme S1.

*Synthesis of **P1***: **M-1** (0.122 g, 0.230 mmol), **M-2** (0.025 g, 0.046 mmol), **M-3** (0.090 g, 0.184 mmol) and $Pd(PPh_3)_4$ (0.028 g, 5% e.q.) were dissolved in the mixture of 14 mL toluene and 9 mL K_2CO_3 solution (2 M). The resulting solution kept stirring for 48 h at 75 ^{o}C in Ar atmosphere and was then cooled to room temperature. After the toluene organic layer was separated, the crude polymer solid was obtained upon evaporation of the solvent under vacuum, which was dissolved in 1 mL CH_2Cl_2 and dropped into 50 mL CH_3OH to stir for 30 min. The produced precipitation was finally filtered and collected as **P1** to afford a yellow polymer solid (0.101 g, 71.2%). ^{1}H NMR (400 MHz, $CDCl_3$) δ 8.40-8.29 (m, 1H), 7.73-7.40 (m, 11H), 7.18-6.99 (m, 11H), 4.36-4.29 (m, 2H), 3.77 (s, 0.6H), 1.89 (s, 1H), 1.63 (s, 4H), 1.33-1.24 (m, 7H), 0.86-0.85 (m, 3H). GPC data: Mw =7460, Mn = 4260, PDI = 1.75.

*Synthesis of **P2**:* **M-1** (0.122 g, 0.230 mmol), **M-3** (0.100 g, 0.204 mmol), **3** (0.014 g, 0.026 mmol) and $Pd(PPh_3)_4$ (0.028 g, 5% e.q.) were dissolved in the mixture of 14 mL toluene and 9 mL K_2CO_3 (2 M). The reaction was proceeded to give **P2** as a green solid (0.094 g, 68.6%). ^{1}H NMR (400 MHz, $CDCl_3$) *δ* 8.26-8.22 (m, 1H), 7.66-7.55 (m, 2H), 7.50-7.24 (m, 6H), 7.14-7.06 (m, 11H), 4.23-4.20 (m, 2H), 1.82-1.80 (m, 1H), 1.56-1.52 (m, 1H), 1.19-1.17 (m, 11H), 0.81-0.76 (m, 3H). GPC data: Mw=7472, Mn=6524, PDI=1.15.

*Synthesis of **P3**:* **M-2** (0.123 g, 0.230 mmol), **10** (0.038 g, 0.230 mmol) and $Pd(PPh_3)_4$ (0.028 g, 5% e.q.) were dissolved in the mixture of 14 mL toluene and 9 mL K_2CO_3 (2 M). The reaction was proceeded to give **P3** as a yellow solid (0.072 g, 75.3%). ^{1}H NMR (400 MHz, $CDCl_3$) *δ* 8.16–7.93 (m, 1H), 7.78–7.24 (m, 11H), 7.13–6.88 (m, 3H), 6.88–6.55 (m, 3H), 3.83–3.60 (m, 3H). GPC data: Mw=8530, Mn=6200, PDI=1.38.

*Synthesis of **P4**:* **M-1** (0.122 g, 0.230 mmol), **M-2** (0.006 g, 0.012 mmol), **M-3** (0.107 g, 0.218 mmol) and $Pd(PPh_3)_4$ (0.028 g, 5% e.q.) were dissolved in the mixture of 14 mL toluene and 9 mL K_2CO_3 (2 M). The reaction was proceeded to give **P4** as a yellow solid (0.096 g, 69.1%). ^{1}H NMR (400 MHz, $CDCl_3$) δ 8.36–8.24 (m, 1H), 7.75–7.61 (m, 3H), 7.59–7.29 (m, 8H), 7.24–6.96 (m, 12H), 4.29 (s, 2H), 1.90–1.83 (m, 1H), 1.65–1.58 (m, 1H), 1.43–1.33 (m, 4H), 1.28–1.23 (m, 6H), 1.27–1.24 (m, 3H). GPC data: Mw=7539, Mn=7504, PDI=1.00.

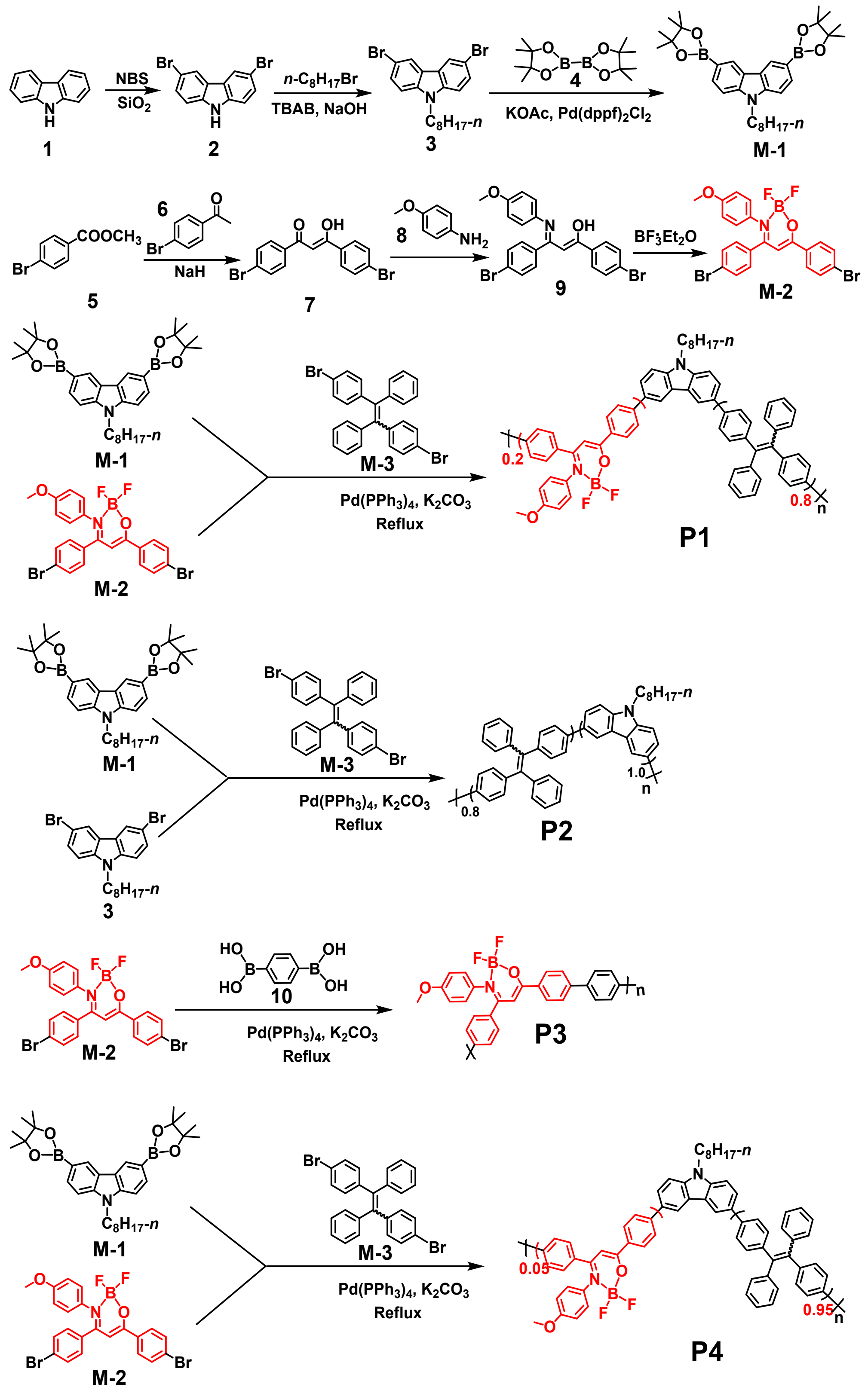

Scheme S1. Synthetic routes of **M-1**, **M-2**, and **P1-P4**.

Synthesis of PCN-224. The PCN-224 nanoparticles were prepared according to previous work.[S4] In brief, H_2TCPP (100 mg, 0.13 mmol), benzoic acid (2.8 g, 23 mmol) and $ZrOCl_2 \cdot 8H_2O$ (300 mg, 0.93 mmol) were dissolved in 140 mL DMF to stir at 300 rpm at 90 °C for 3 h. The resulting PCN-224 nanoparticles were collected by centrifugation (15000 rpm, 30 min) followed by washing with fresh DMF for three times, which were then suspended in DMF at 4°C for further characterization and analysis. Prior to use, the nanoparticles were washed with water.

Preparation of Pdots and Pdots-DNA. Pdots were prepared in aqueous solution through nanoprecipitation according to a previous work.[S5] In brief, the mixture containing 50 μg mL^{-1} polymer and 10 μg mL^{-1} PSMA was firstly prepared in 1 mL THF. After ultrasonically degassed for 20 minutes, the mixture was quickly added to 9 mL water while sonicating for 4 min. The THF was removed by rotary evaporation under vacuum followed by filtration through a 0.22 μm poly(ether sulfones) syringe filter to obtain carboxyl Pdots dispersion.

The Pdots dispersion (0.15 mg mL^{-1}, 500 μL) was mixed completely with HEPES buffer (1 M, 6 μL) and PEG (5% w/v, Mw 3350, 6 μL), and the pH was adjusted to 7.1. After that, 60 μL cDNA (1 μM) and 60 μL EDC (5 mg mL^{-1}) were added to the mixture and vibrated for 3 h at room temperature. The resulting Pdots-DNA was separated by ultrafiltration for three times to remove free cDNA.

Preparation of Pdots@PCN-224 Probe. The PCN-224 nanoparticles were functionalized with Pdots-cDNA by electrostatic adsorption. Firstly, 100 μL electropositive PCN-224 (1.0 mg mL^{-1}) was mixed with 500 μL electronegative Pdots-cDNA and gently shocked for 4 h. After washing with water for three times. The resulting solution was centrifugated with 1×PBS (pH 7.4) and stored at 4 °C for further use.

Polyacrylamide Gel Electrophoresis (PAGE) Analysis. The mixture of 5 μL DNA and 1.5 μL 6×loading buffer was prepared to perform 15% PAGE analysis by running at 100 V for 60 min, and then staining with SYBR gold nucleic acid gel stain (1×concentrate in TBE buffer) for 30 min. Bio-Rad ChemDoc XRS facility was used to obtain the resulting boards.

Cell Culturing. MCF-7 cells and MDA-MB-231 cells were cultured in RPMI-1640 media supplemented with 10% FBS, streptomycin (0.1 mg mL^{-1}) and penicillin (0.1 mg mL^{-1}). HepG2 cells and NB-4 cells were cultured in DMEM supplemented with 10% FBS, streptomycin (0.1 mg mL^{-1}),

and penicillin (0.1 mg mL^{-1}). SK-BR-3 cells were cultured in McCoy's 5A media supplemented with 10% FBS and 1% penicillin-streptomycin solution (0.1 mg mL^{-1}). Cells were grown at 37 °C in a humidified atmosphere containing 5% CO_2 and maintained at densities between 5×10^5 and 2×10^6 cells mL^{-1}. Prior to use, 10% goat serum was added to block cell surface at 37 °C for 30 min.

CLSM Imaging of Apt1 Binding to MUC1 and HER2 on Cell Surface. MCF-7 (MUC1-positive) and SK-BR-3 cells (HER2-positive) were separately seeded on four-well confocal dishes and cultured overnight and 3 days, respectively. After washing for three times with PBS, cells were blocked with 10% goat serum to block cell surface at 37 °C for 30 min. After washing for three times with PBS, the cells were incubated at 4 ^oC with Random 1-FAM (1.0 μM) or Apt1'-FAM (1.0 μM) for MUC1 cells, and Random 2-FAM (1.0 μM) or Apt1''-FAM (1.0 μM) for SK-BR-3 cells for 30 min, respectively, followed by washing with PBS for three times and the emission signals of FAM on cell surface were imaged with CLSM from 495 to 545 nm under 488 nm excitation.

Exo III-Assisted Recycling Assay. The mixture (20 μL) containing Apt1' (0.2 μM), Apt1'' (0.2 μM), HP2' (0.2 μM), HP2'' (0.2 μM), Exo III (5 U), and different concentrations of MUC1 or HER2 in 1×PBS (pH 7.4) was incubated for 1 h at 37 °C. The solution was then heated at 80 °C for 10 min to deactivate Exo III and prevent the digestion reaction. The obtained target recycling solution was used for ECL imaging at a HP3/MCH/Au/ITO electrode. During the cell detection, 2000 cells were added into the mixture instead of the protein.

Fabrication of HP3/MCH/Au/ITO Electrode. The Au/ITO (5 nm chrome followed by 50 nm gold) slide was fabricated with electron beam evaporation technique (DE Tec. Ltd, USA), and pasted with a porous sticker with well array as working electrode array. Equal volumes of annealed HS-HP3 (10 μM) and TCEP (1 mM) were mixed for 1 h to reduce disulfide bond, following a dilution with PBS to get HS-HP3 at 1.5 μM. 2.5 μL of HS-HP3 (1.5 μM) was dropped on electrode surface to incubate at 37 ^oC for 2 h. After rinsing with PBS (pH 7.4) and drying with nitrogen, 2.5 μL of MCH (1 mM) was dropped on electrode to incubate for 1 h for blocking the unmodified sites. After washing with PBS and drying with nitrogen, the electrochemical DNA array (HP3/MCH/Au/ITO) was obtained and stored at 4 ^oC for future use.

Measurement Procedure. Prior to measurement, 2.5 μL of above target recycling mixture was dropped onto the surface of HP3/MCH/Au/ITO and kept at 37 °C for 1 h to capture the released S-DNA. Finally, 2.5 μL Pdots@PCN-224 probe solution was dropped onto the surface for further hybridization at 37 °C for 1.5 h. The signal probe was thus loaded, and the array was dried at 37 °C for 30 min to perform the ECL imaging detection. The electrode was rinsed three times with PBS buffer after each hybridization step.

ECL Imaging Detection. The ECL imaging system equipped with a classical three-electrode system containing the modified Au/ITO as working electrode, a platinum wire as counter electrode, and a Ag/AgCl electrode as the reference electrode by applying a constant potential of +1.0 V for 3 s in 0.1 M pH 7.4 PBS containing 0.1 M KNO_3 and 25 mM TPrA solution.

ECL Spectra. After 200 μL of 50 μg mL^{-1} Pdots or 1 mM $Ru(bpy)_3^{2+}$ was coated at carbon/ITO electrode, the modified electrode was immersed in 0.1 M pH 7.4 PBS containing 0.1 M KNO_3 and 0.1 M TPrA to apply the potential of +1.1 or +1.2 V for 9 s. CHI 660D electrochemical workstation conjugating with F-7000 fluorescence spectrometer under the closed shutter was used to record the ECL spectra of Pdots and $Ru(bpy)_3^{2+}$. The relative ECL efficiency was calculated using the equation:

$$\Phi_{ECL} = \Phi°_{ECL}(I/Q)/(I°/Q°) \qquad (1)$$

where Q and $Q°$ are the consumed charges (integrating current vs time), I and $I°$ are the integrated ECL intensities (integrating ECL spectrum vs wavelength), and Φ_{ECL} and $\Phi°_{ECL}$ are the ECL efficiency of the sample and standard, respectively.[S6] In this work, 1 mM $Ru(bpy)_3^{2+}$ was used as the standard with the $\Phi°_{ECL}$ value of 1.

Supplementary Figures

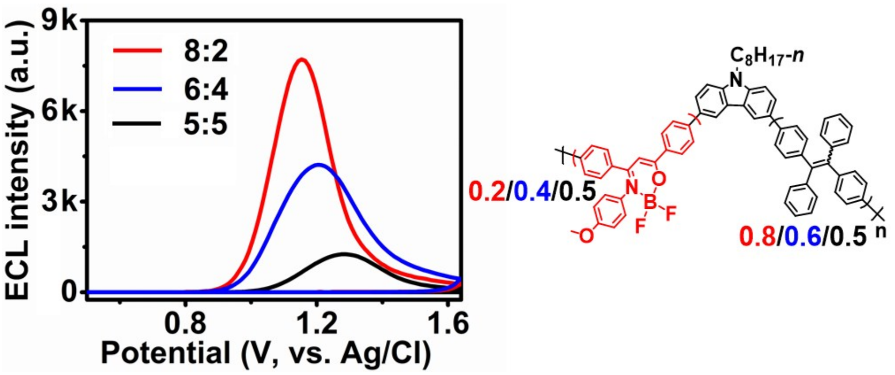

Fig. S1 ECL curves of Pdots with different radios of A1/D2 to A2 in 0.1 M PBS (pH 7.4) containing 0.1 M KNO_3 and 25 mM TPrA. PMT: 300 V.

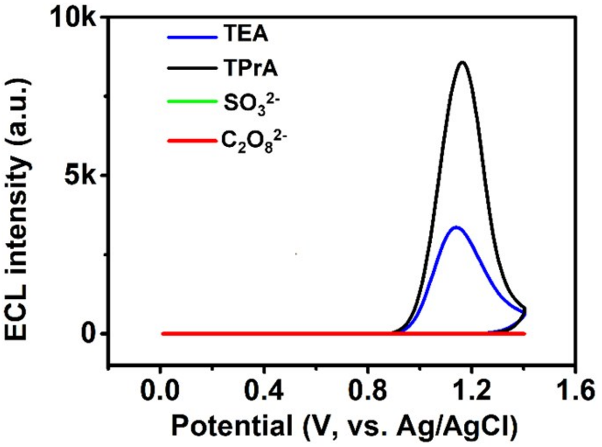

Fig. S2 ECL curves of **P1** dots|GCE in 0.1 M PBS (pH 7.4) containing 0.1 M KNO_3 and 25 mM $K_2C_2O_4$, Na_2SO_3, TEA or TPrA. PMT: 300 V.

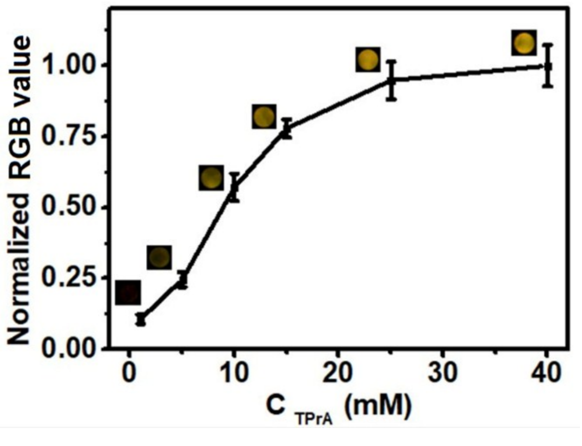

Fig. S3 Normalized intensity of **P1** dots in 0.1 M PBS containing 0.1 M KNO_3 and different concentration of TPrA for ECL imaging (insets).

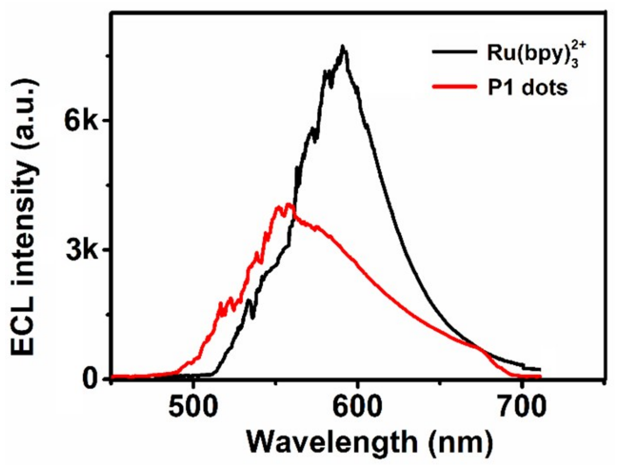

Fig. S4 ECL spectra of 50 μg mL^{-1} **P1** dots and 1 mM $Ru(bpy)_3^{2+}$ modified carbon/ITO electrodes in 0.1 M PBS (pH 7.4) containing 0.1 M TPrA.

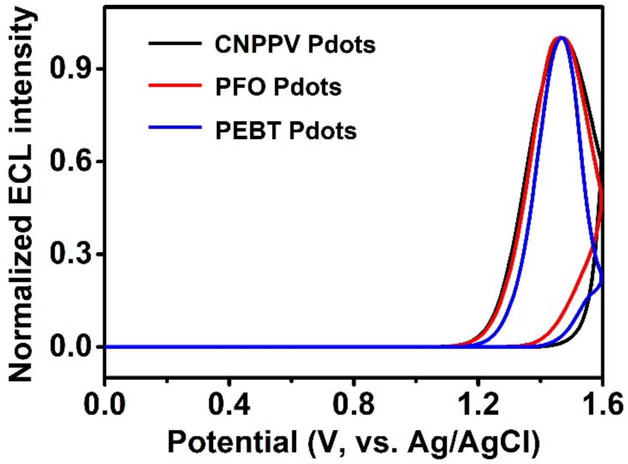

Fig. S5 ECL curves of CNPPV Pdots, PFO Pdots and PFBT Pdots|GCE in 0.1 M PBS (pH 7.4) containing 25 mM TPrA.

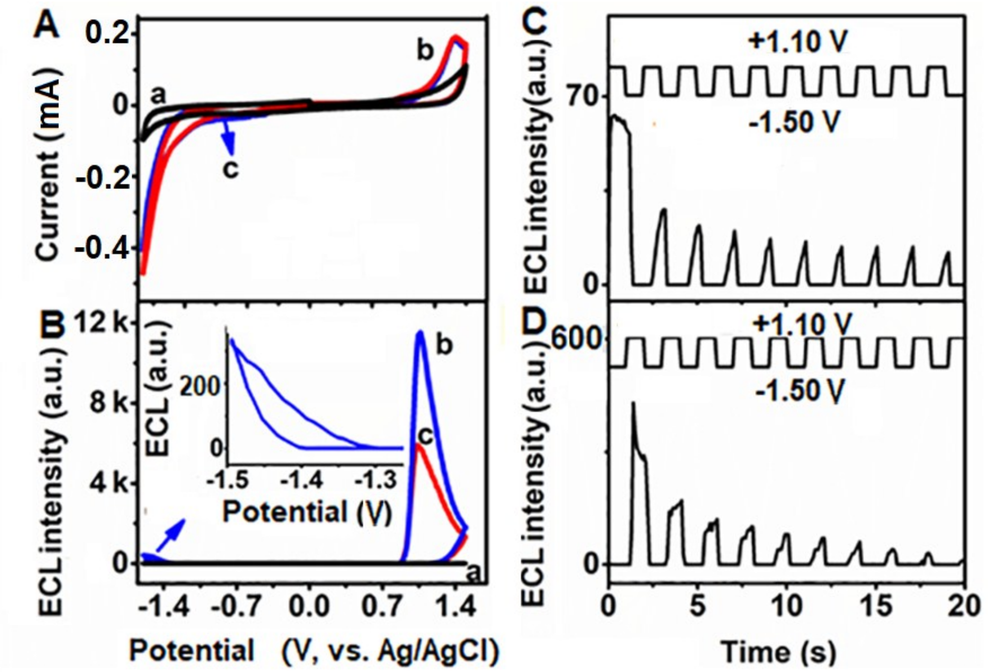

Fig. S6 (A) CV and (B) ECL curves of bare (a) and **P1** dots|GCE (b, c) in nitrogen-saturated 0.1 M pH 7.4 PBS from 0 to −1.50 and then +1.50 V (b), and 0 to +1.50 and then −1.50 V (a, c) at 100 mV s^{-1}. ECL transients of **P1** dots|GCE in nitrogen-saturated 0.1 M pH 7.4 PBS by multipotential steps of 1 s at (C) +1.10 to −1.50 V, and (D) -1.50 V to +1.10 V. PMT: 500 V.

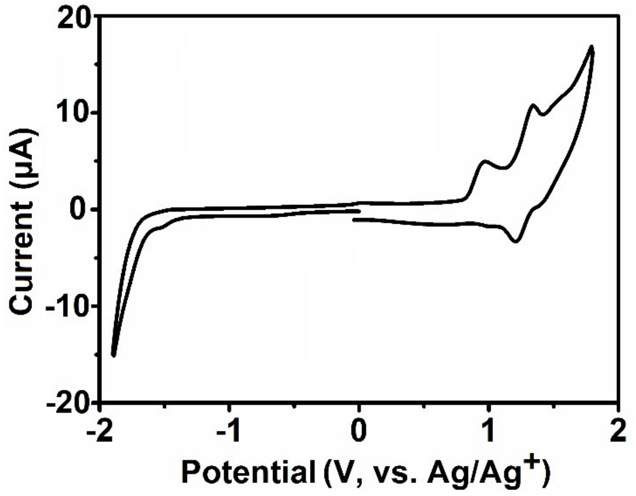

Fig. S7 CV curve of **P1** measured in CH_2Cl_2 solution containing 0.1 M Bu_4NBF_4. Scan rate: 100 mV s^{-1}.

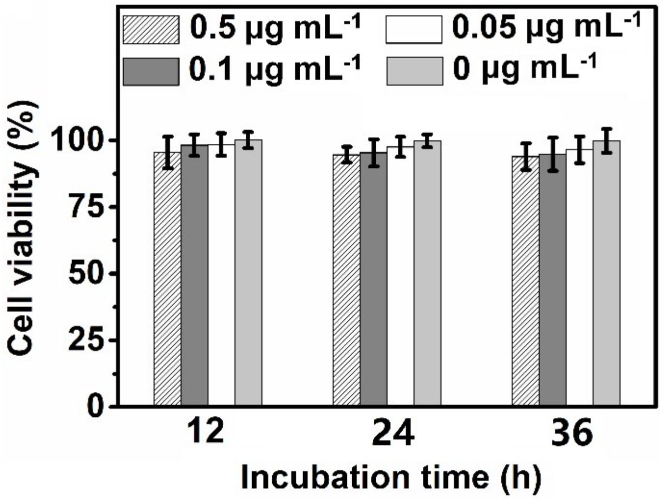

Fig. S8 Metabolic viability of MCF-7 cells after incubation with **P1** dots suspension at different concentrations for 12 h, 24 h and 36 h.

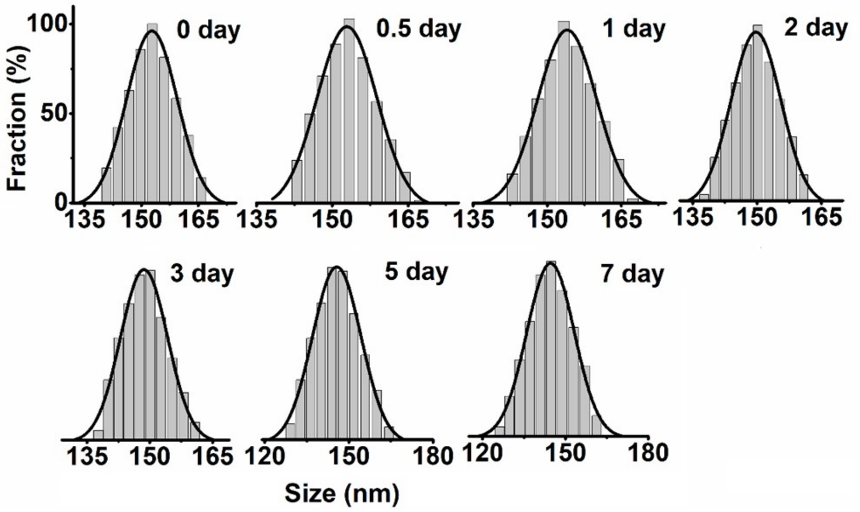

Fig. S9 DLS characterization of probe after stored at 4 °C for 0-7 days.

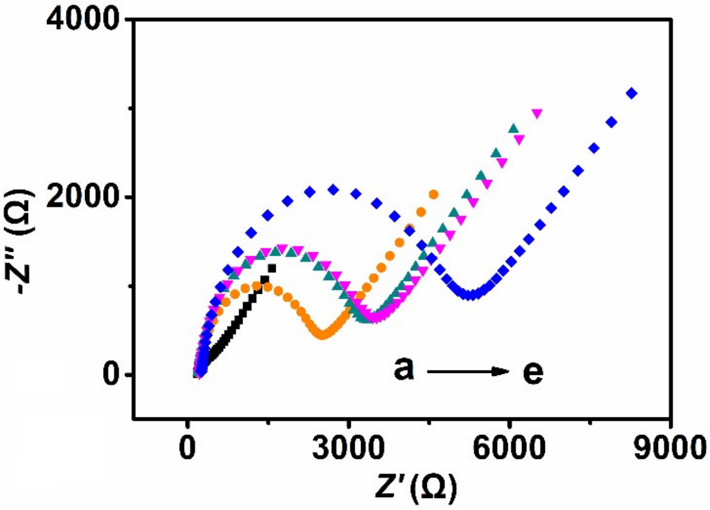

Fig. S10 EIS of Au/ITO (a), HP3/Au/ITO (b), HP3/MCH/Au/ITO (c), and HP3/MCH/Au/ITO + probe in absence (d) and presence (e) of target recycling mixture.

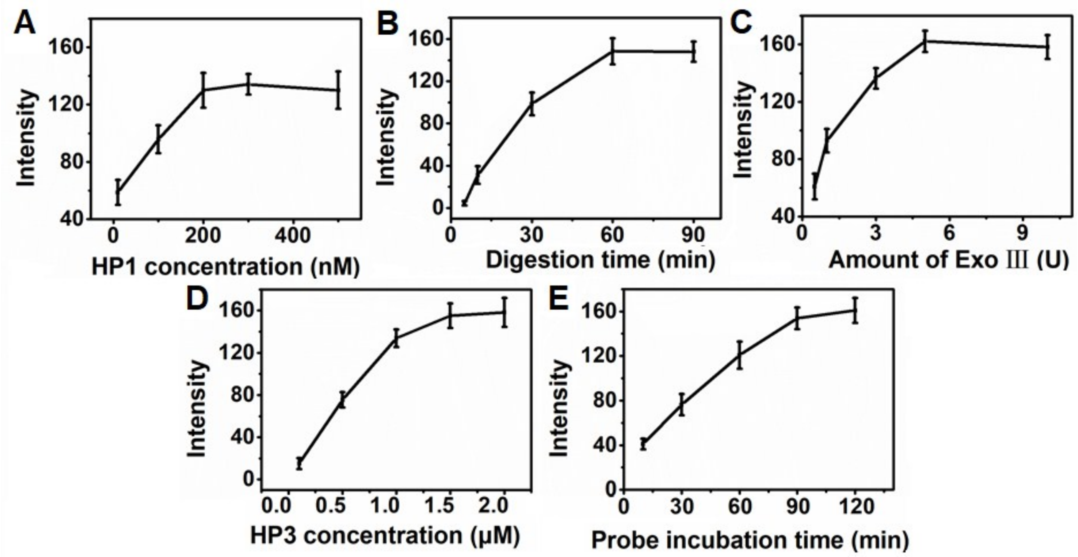

Fig. S11 Effects of (A) Apt1 concentration, (B) digestion time with Exo III, (C) Exo III amount, (D) HP3 concentration, and (E) incubation time with probe for MUC1 at 5 ng mL^{-1} on intensity of ECL imaging (n = 3).

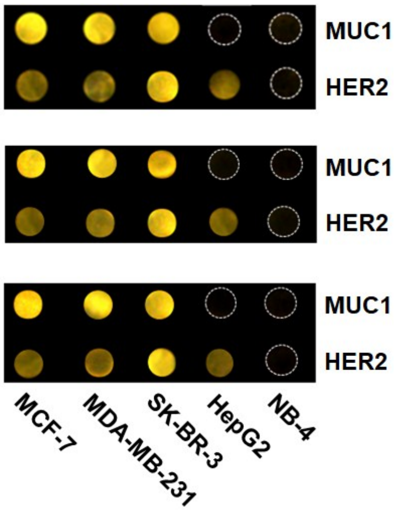

Fig. S12 ECL images of parallel experiments to detect MUC1 and HER2 on different cells (n = 3).

Supporting Tables

Table S1. Comparison of ECL efficiency of various triple-component Pdots.

Polymer	Standard control (co-reactant)	ECL efficiency	Reference
A	20 μg mL^{-1} Pdots vs. 1 μM $Ru(bpy)_3^{2+}$ (25 mM TPrA)	11%	[S7]
B	50 μg mL^{-1} Pdots vs. 1 mM $Ru(bpy)_3^{2+}$ (100 mM TPrA)	11.8%	[S8]
C	50 μg mL^{-1} Pdots vs. 1 mM $Ru(bpy)_3^{2+}$ (100 mM TPrA)	5.8%	[S8]
D	50 μg mL^{-1} Pdots vs. 1 mM $Ru(bpy)_3^{2+}$ (100 mM TPrA)	23.1%	This work

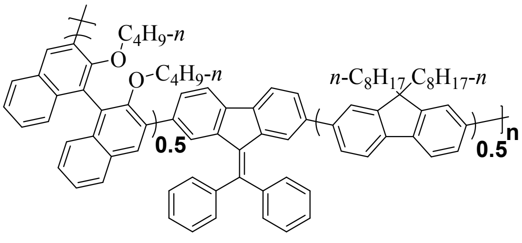

A

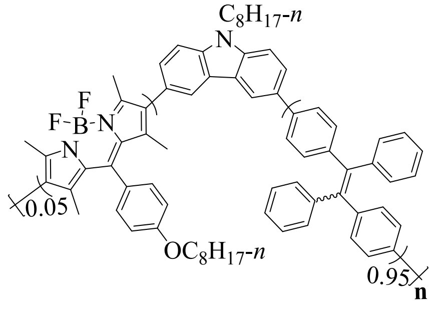

B

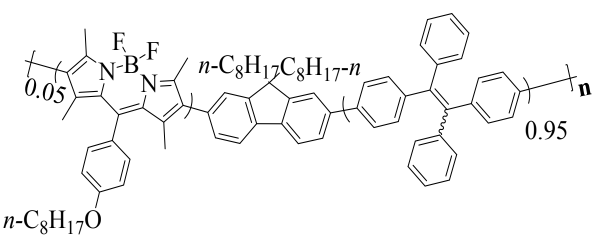

C

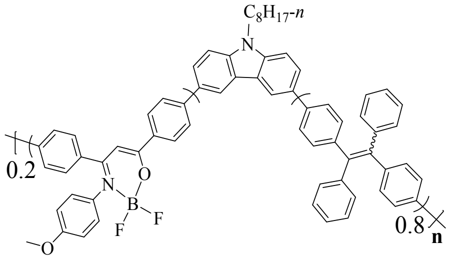

D

Table S2. Anodic ECL behaviors of **P1** dots vs. commercial Pdots.

Pdots	Reaction conditions	ECL onset potential	ECL peak potential	Reference
PFBT NPs/TPrA	0.1 M $LiClO_4$ in MeCN	+1.6 V	+1.8 V	[S9]
PFO dots/$C_2O_4^{2-}$	0.1 M pH 6.5 in PBS	+0.7 V	+1.9 V	[S10]
CNPPV dots/TPrA	0.1 M pH 7.4 in PBS	+1.1 V	+1.4 V	[S11]
**P1** dots/TPrA	0.1 M pH 7.4 in PBS	+0.89 V	+1.12 V	This work

Table S3. DNA sequence information.

Protein	Name	DNA Sequence (5'-3')
MUC1	Apt1'	GCAGTTGATCCTTTGGATACCCTGGTTTCGTGGATCAACTG CTTCTAGGA
	Hairpin 2' (HP2')	TCAACTGCTTCTAGGCTTGAGGATGAAGCAGTTGATCCACG
	Hairpin 3' (HP3')	TCAACTGCTTCTAGGCCACTTTTACCTCAAGCCTAGAAGCA GTTGATTT-SH
	Capture DNA (cDNA 1')	GTGGCCTAGAAGCAGTTGATTTTTT-NH_2
	Random 1	CCCAGAAATTCTTGCAACAATTTTTTGAACTTCGAATTTCTG GGTTAAGG
HER2	Apt1''	GCAGCGGTGTGGGGGCAGCGGTGTGGGGGCAGCGGTGTG GGGTTTTTTTGGCACACCGCTGCATACGG
	Hairpin 2'' (HP2'')	ACACCGCTGCATACGGGGCACTTTTGCAGCGGTGTGCCAA A
	Hairpin 3'' (HP3'')	ACACCGCTGCATACGGCCGTGAAGTGCCCCGTATGCAGCG GTGTTTT-SH
	Capture DNA (cDNA 1'')	ACGGCCGTATGCAGCGGTGTTTTTTT-NH_2
	Random 2	CCCAGAAATTCTTGCAACAATTTTTTTTTTTTTTTTGAACTTT TTTTGCAAGAATTTCTGGGTTAAGG

*All DNAs were annealed at 95 ^{o}C for 5 min and slowly cooled down to room temperature, and stored at 4 ^{o}C prior to use.

Table S4. Comparison of the proposed method with those reported for MUC1 and HER2.

Protein	Detection limit	Method	Linear range	Reference
MUC1	2.2 nM	Electrochemistry	8.8-353.3 nM	[S12]
	0.25 pM	Electrochemistry	0.001-1000 nM	[S13]
	0.3 nM	Photoelectrochemistry	1-5000 nM	[S14]
	40 nM	ECL	64.9-1036.8 nM	[S15]
	5 pg mL^{-1}	ECL	0.005-1000 ng mL^{-1}	[S16]
	1 pg mL^{-1} (5 fM)	ECL imaging	0.001-5 ng mL^{-1}	This work
HER2	6 ng mL^{-1}	Electrochemistry	0-40 ng mL^{-1}	[S17]
	12 pg mL^{-1}	Electrochemistry	0.02-12.5 ng mL^{-1}	[S18]
	210 pg mL^{-1}	Electrochemistry	0.5-75 ng mL^{-1}	[S19]
	26 pg mL^{-1}	Electrochemistry	0.1-32 ng mL^{-1}	[S20]
	10 pg mL^{-1}	Electrochemistry	0.01-100 ng mL^{-1}	[S21]
	5 pg mL^{-1}	ECL imaging	0.005-10 ng mL^{-1}	This work

Annex

^{1}H NMR for **P1**

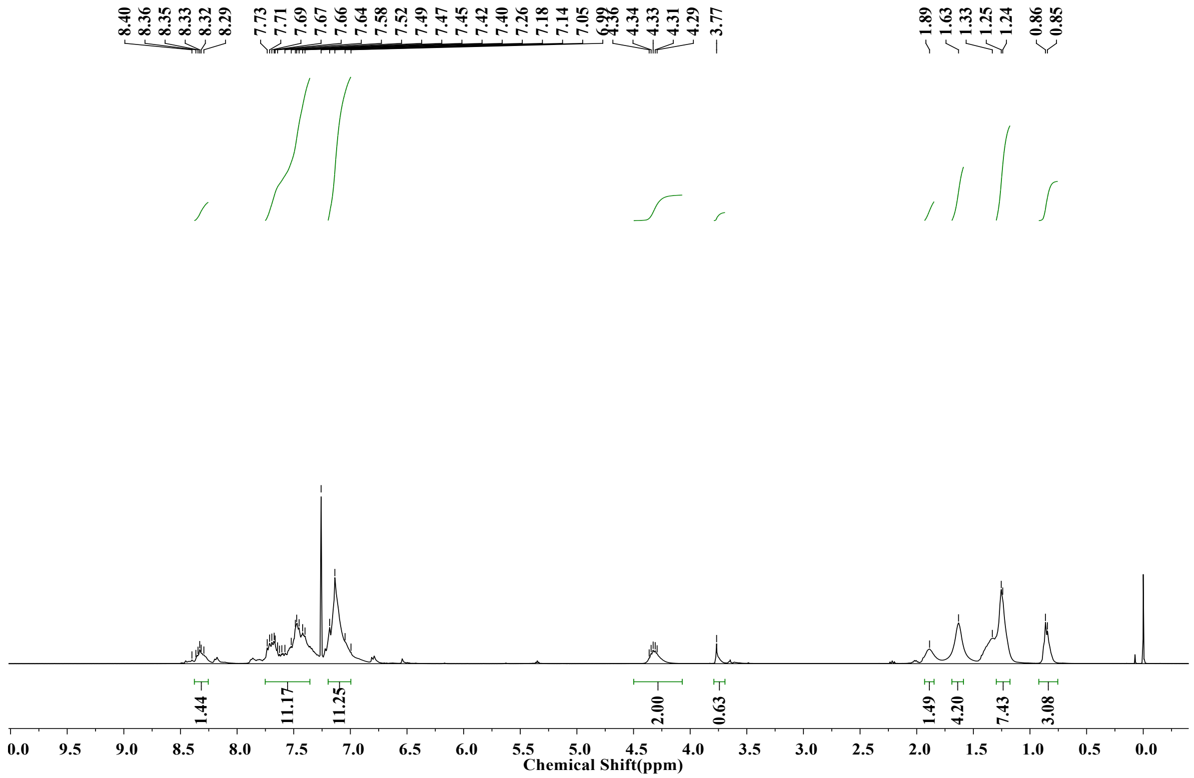

^{1}H NMR for **P2**

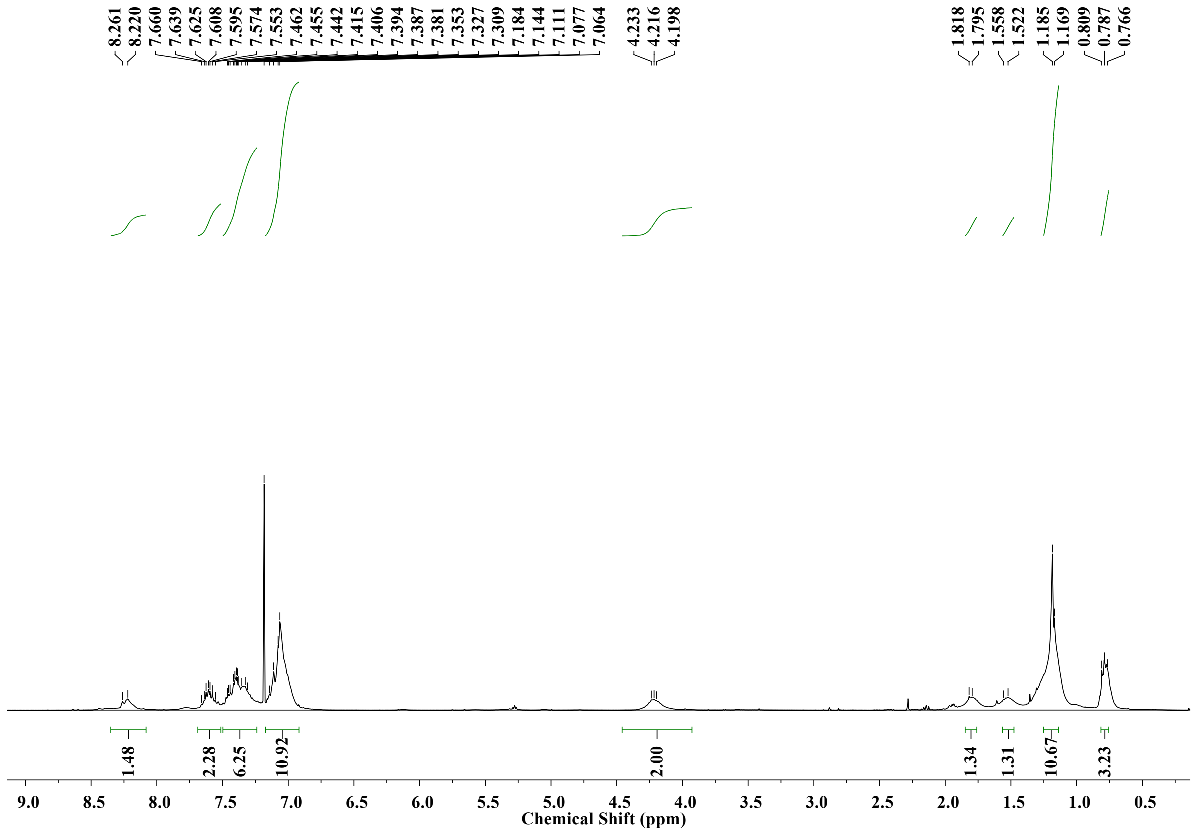

^1H NMR for **P3**

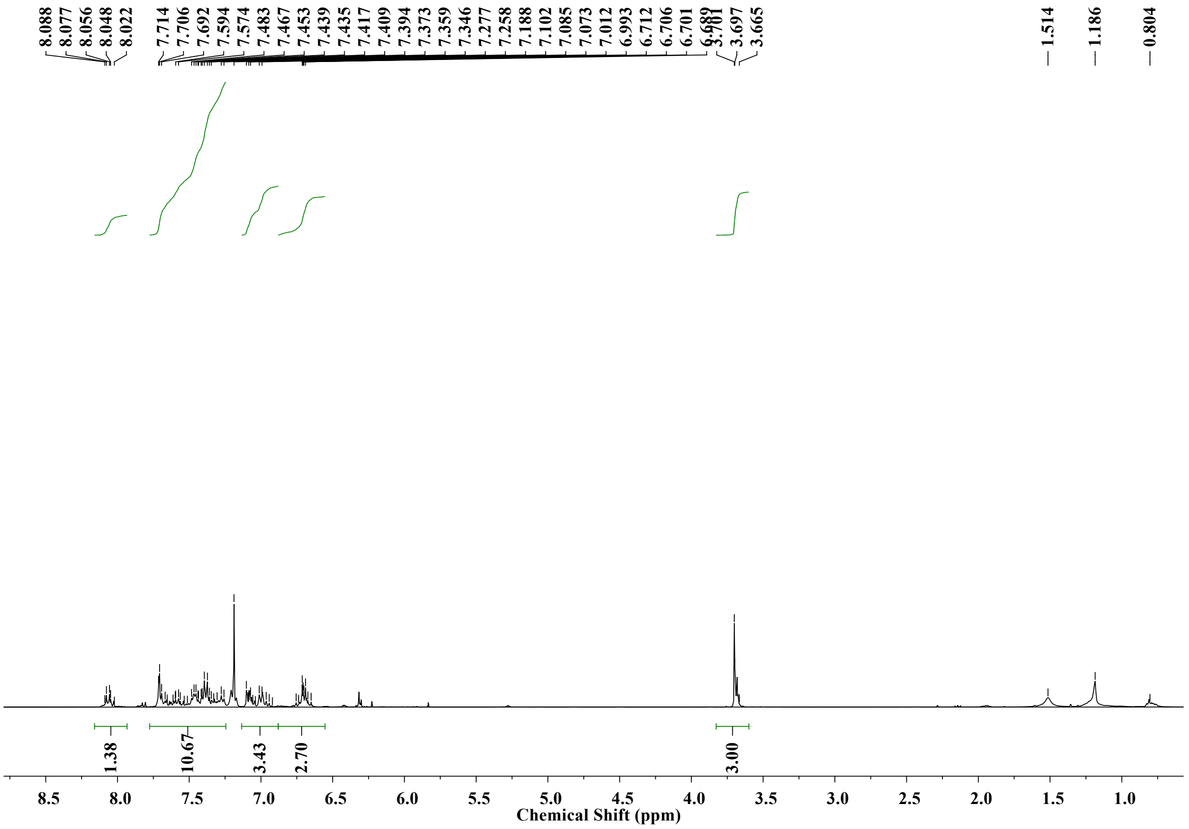

^1H NMR for **P4**

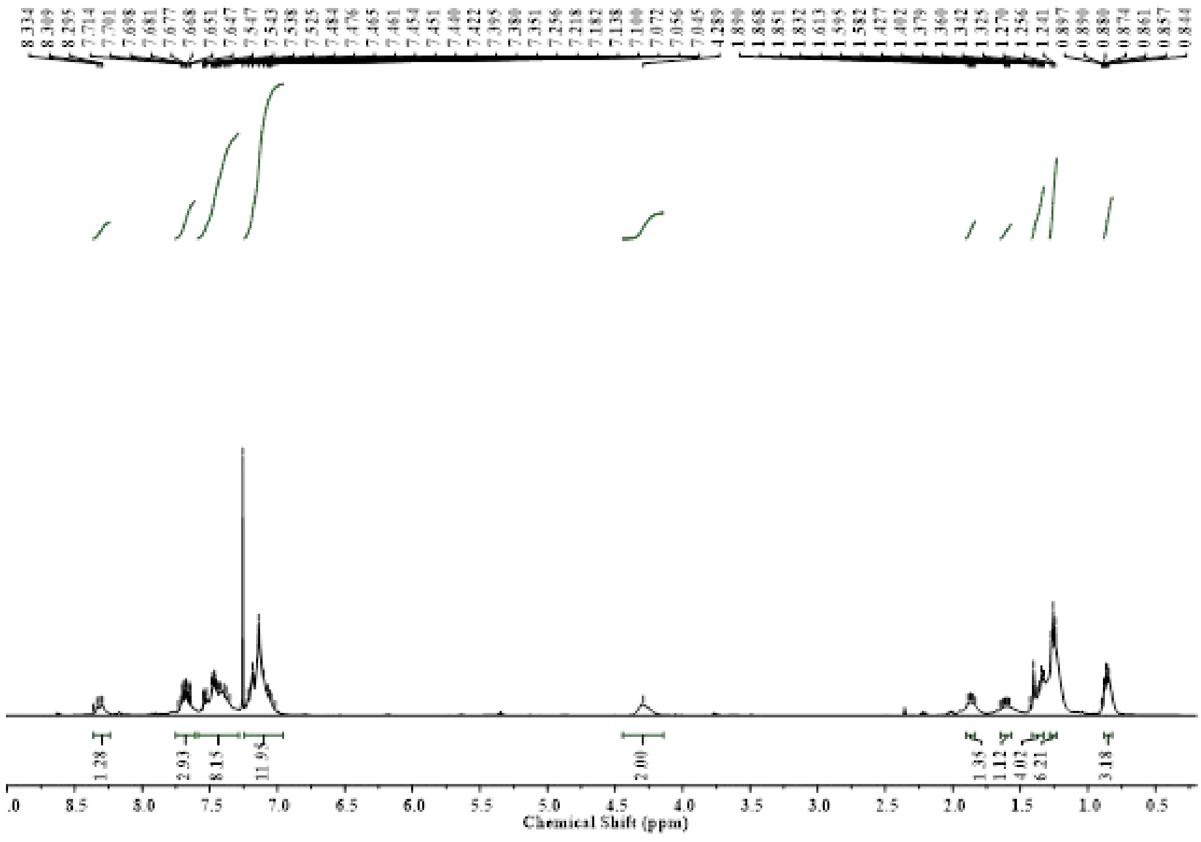

Supporting References

[S1] L. Zhang, S. H. Zeng, L. X. Yin, C. Y. Ji, K. C. Li, Y. Q. Li and Y. Wang, *New J. Chem.*, 2013, **37**, 632−639.

[S2] S. W. Zhang, Y. Sheng, G. Wei, Y. W. Quan, Y. X. Cheng and C. J. Zhu, *Polym. Chem.,* 2015, **6**, 2416−2422.

[S3] C. H. Dai, D. L. Yang, X. Fu, Q. M. Chen, C. J. Zhu, Y. X. Cheng and L. H. Wang, *Polym. Chem.*, 2015, **6**, 5070−5076.

[S4] J. Park, Q. Jiang, D. W. Feng, L. Q. Mao and H. C. Zhou, *J. Am. Chem. Soc.*, 2016, **138**, 3518−3525.

[S5] C. Wu, S. J. Hansen, Q. Hou, J. Yu, M. Zeigler, Y. Jin, D. Burnham, J. McNeill, J. Olson and D. T. Chiu, *Angew. Chem. Int. Ed.*, 2011, **50**, 3430−3434.

[S6] M. Hesari, M. S. Workentin and Z. F. Ding, *ACS Nano,* 2014, **8**, 8543−8553.

[S7] F. Sun, Z. Y. Wang, Y. Q. Feng, Y. X. Cheng, Y. W. Quan and H. X. Ju, *Biosens. Bioelectron*., 2018, **100**, 28−34.

[S8] Z. Y. Wang, Y. Q. Feng, N. N. Wang, Y. X. Cheng, Y. W. Quan and H. X. Ju, *J. Phys. Chem. Lett.*, 2018, **9**, 5296−5302.

[S9] Y. L. Chang, R. E. Palacios, F. R. F. Fan, A. J. Bard and P. F. Barbara, *J. Am. Chem. Soc.*, 2008, **130**, 8906−8907.

[S10] Q. Y. Lu, J. J. Zhang, Y. Y. Wu and S. H. Chen, *RSC Adv.*, 2015, **5**, 63650−63654.

[S11] Y. Q. Feng, N. N. Wang and H. X. Ju, *Anal. Chem*., 2018, **90**, 1202−1208.

[S12] R. Hu, W. Wen, Q. L. Wang, H. Y. Xiong, X. H. Zhang, H. S. Gu and S. F. Wang, *Biosens. Bioelectron.*, **2014**, *53*, 384–389.

[S13] S. H. Yang, F. F. Zhang, Q. L. Liang and Z. H. Wang, *Biosens. Bioelectron.*, 2018, **120**, 85–92.

[S14] X. P. Liu, P. P. Li, C. J. Mao, H. L. Niu, J. M. Song and B. K. Jin, *Sens Actuators B Chem.*, 2018, **275**, 251–259.

[S15] W. Wei, D. F. Li, X. H. Pan and S. Q. Liu, *Analyst*, 2012, **137**, 2101–2106.

[S16] N. Zhang, W. R. Li, Z. Y. Guo, Y. H. Sha, S. Wang, X. R. Su and X. H. Jiang, *Electroanalysis*, 2016, **28**, 1504–1509.

[S17] A. Ravalli, C. G. da Rocha, H. Yamanaka and G. Marrazza, *Bioelectrochemistry*, 2015, **106**, 268−275.

[S18] S. Carvajal, S. N. Fera, A. L. Jones, T. A. Baldo, I. M. Mosa, J. F. Rusling and C. E. Krause, *Biosens. Bioelectron.*, 2018, **104**, 158–162.

[S19] A. Tabasi, A. Noorbakhsh and E. Sharifi, *Biosens. Bioelectron.*, 2017, **95**, 117–123.

[S20] U. Eletxigerra, J. Martinez-Perdiguero, S. Merino, R. Barderas, R. M. Torrente-Rodríguez, R. Villalonga, J. M. Pingarrón and S. Campuzano, *Biosens. Bioelectron.*, 2015**, 70**, 34–41.

[S21] S. Sharma, J. Zapatero-Rodríguez, R. Saxena, R. O'Kennedy and S. Srivastava, *Biosens. Bioelectron.*, 2018, **106**, 78–85.
